# A Rare Case of Splenic Infarct Secondary to Mobile Cardiac Echodensity

**DOI:** 10.7759/cureus.46434

**Published:** 2023-10-03

**Authors:** Sruthi Ramanan, Harjinder Singh, Omair Ahmed, Mark Zande, Malcom Trimble

**Affiliations:** 1 Internal Medicine, Henry Ford Health System, Jackson, USA; 2 Cardiology, Henry Ford Health System, Jackson, USA; 3 Hematology Oncology, Henry Ford Health System, Jackson, USA

**Keywords:** oral anticoagulation, transesophageal echo, aortic echodensity, cardiac embolism, splenic infarct, lambl

## Abstract

Lambl’s excrescences (LE) are mobile filiform lesions, mostly found on the left-sided heart valves. Histologically, they have a mesenchymal origin with a single endothelial layer. They have the potential to detach, resulting in catastrophic thromboembolic events. Their rarity often leads to them being misdiagnosed as vegetations of endocarditis with patients failing to improve on conventional therapy.

A 48-year-old female with a history of hypertension presented to the Emergency Department with a one-week history of sharp left upper quadrant pain. She was vitally stable; the only lab abnormality was revealed to be a mildly elevated white cell count. CT abdomen revealed a splenic infarct involving 25% of the parenchyma. Patients had no history of abdominal trauma, coagulation disorders, exogenous estrogen use or IV drug abuse. Subsequent investigations failed to reveal any cause of hypercoagulability. An extensive cardiac workup revealed no arrhythmias, but transesophageal echocardiogram showed a mobile echo density on the ventricular side of the aortic valve attached at the coaptation zone, approximately 2.7 cm long and 0.1 cm wide, suggesting a very prominent Lambl’s excrescence. In the absence of any other findings, the patient’s splenic infarct was determined to be secondary to an embolic event from the aortic valve lesion. Rivaroxaban was initiated and the patient subsequently improved.

Existing literature describes most LEs as being asymptomatic and discovered incidentally on echocardiograms. This case illustrates the need to develop a criterion for prompt identification of LEs and differentiating them from the vegetations of endocarditis. It also brings forth the question of prophylactic treatment of these lesions while highlighting the lack of guidelines regarding the management of embolic phenomena secondary to LE.

## Introduction

Lambl’s excrescences (LE) were first described by Vilém Dušan Lambl in 1856 [1]. These are mobile, fine, filiform lesions that are found at areas of cardiac valve closure. The extensions are commonly associated with left-sided heart valves (mitral and the aortic) [2-4]. The LEs can detach and migrate via the blood vessels causing catastrophic thromboembolic events in patients. They have been associated with ischemic/embolic stroke and acute coronary syndromes [5,6]. These lesions are commonly identified on echocardiography. Vegetations caused by endocarditis, papillary fibroelastoma and aortic valve fenestration are a few conditions that are commonly mistaken for Lambl’s excrescences [7]. Herein, we report a case of Lambl’s excrescences causing splenic thrombosis.

## Case presentation

A 48-year-old lady with a history of hypertension and smoking presented to the Emergency Department complaining of sharp left upper quadrant abdominal pain for one week. Patient was hemodynamically stable, afebrile and saturating well on room air. Physical examination demonstrated tenderness of the left upper quadrant. Labs showed mild leukocytosis and a normal complete metabolic profile.

**Figure 1:**
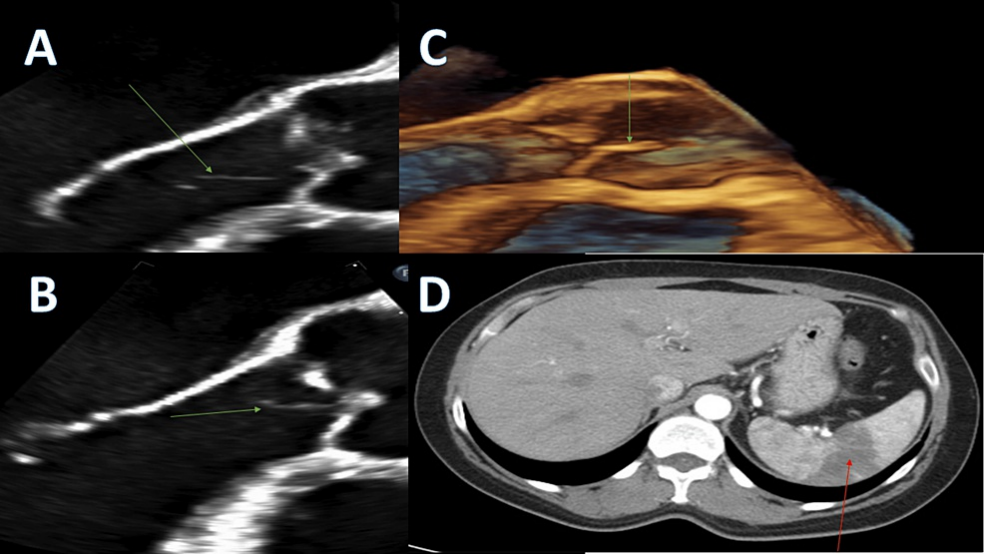
Images of vegetation and splenic infarct. A and B: Green arrow pointing to aortic vegetation on TEE, C: Green arrow pointing to aortic vegetation on TEE with reconstruction, D: Red arrow pointing to homogenous opacity on spleen indicating infarcted tissue TEE: transesophageal echocardiogram

Computed tomography (CT) abdomen revealed splenic infarct (Figure 1) involving 25% of the splenic parenchyma. Patient denied abdominal trauma, thromboembolism, or exogenous estrogen use. No other cause of hypercoagulability was identified.

She underwent extensive cardiac workup to eliminate an embolism of cardiac origin as the cause of splenic infarction. 30-day Holter monitor did not show any evidence of atrial fibrillation or other arrhythmia. Transesophageal echocardiogram (TEE) showed mobile echodensity on the ventricular side of the aortic valve attached at the coaptation zone, 2.7 cm long and 0.1 cm wide, suggesting very prominent Lambl’s excrescence (as seen in Figure 1A, 1B, 1C), and an otherwise unremarkable exam.

Laboratory workup showed normal levels of beta-2 glycoprotein antibodies and normal cardiolipin antibodies. Autoimmune workup showed anti-nuclear antibody (ANA) titre of 1:80 (speckled), negative rheumatoid factor and normal complement levels. Blood smear showed normal red blood cell (RBC) morphology. Hepatitis C virus (HCV) antibodies were normal. Embolic event from the aortic valve lesion was the most plausible explanation for the patient's splenic infarction. Rivaroxaban was initiated for management.

## Discussion

LEs are often asymptomatic and identified incidentally during routine echocardiograms. They can often be found on valve closure lines as a papillary extension [6]. These have a varied appearance and can be found as single strands, rows, or clusters. Histologically they are extensions of the connective tissue layer of the valve covered with a single layer of endothelium [8].

Our patient’s lesion was present on the aortic valve like the case described by Lambl et al. [1], however Magarey et al. demonstrated the prevalence of LE to be 85% in mitral valve and 2% in aortic valve based on autopsy findings [9]. Magarey et al.’s findings were further supported by Freedberg [10] and Roldan [11] as they found LE more often in the mitral valve.

The pathogenesis behind development of these lesions seems to be repeated trauma from the passage of blood causing microtears in the endothelium. This results in fibrin deposition and fibrous scar formation that is later covered by an endothelial layer [6].

Echocardiogram is used to identify the lesions, with TEE being favored over transthoracic echo (TTE). However even with TEE the sensitivity and specificity are low at 68% and 85% respectively [8]. This is due to lack of consensus on identification of mobile echodensities in echocardiograms.

LEs are often mistaken for vegetations caused by endocarditis, papillary fibroelastoma and aortic valve fenestration [7]. Histopathological examination helps to differentiate these conditions.

LEs are rarely associated with ischemic stroke [3,8,12-14] and acute coronary syndrome [15,16], however there is a paucity of literature regarding the association of LE with splenic thrombosis. There has been one reported case of LE with embolus to the popliteal artery [17].

Given the rarity of this phenomenon, there are no guidelines on treatment of patients with LE. Currently based on the available case reports treatment varies from antiplatelet therapy with single or dual agents in patients with single embolic phenomenon [18-20]. In cases with recurrent embolic phenomenon, the standard of treatment tends to be either anticoagulants or surgery [3,13,14].

## Conclusions

In conclusion, existing literature reports an association between the presence of LE to ischemia/embolic events. However, there is scant evidence for the clinical significance of the presence of these lesions. A paucity in the literature has made it difficult to determine the guidelines for management of patients with such ischemic/embolic events. There is a need to determine the clinical relevance of these lesions and their management.
